# The principle of pooled calibrations delivers full correspondence between uncertainties of measurements of Na, Mg and Ni when determined using HR-CS FAAS

**DOI:** 10.1016/j.heliyon.2023.e13562

**Published:** 2023-02-08

**Authors:** Jens E.T. Andersen, Keaboletse Moemedi, Kebabonye Katse

**Affiliations:** Botswana International University of Science and Technology, Department of Chemical and Forensic Sciences, Plot 10071, Boseja Ward, Private Bag 016, Palapye, Botswana

**Keywords:** Quality assurance, Quality control, High-resolution continuum-source atomic absorption spectrometry, Principle of pooled calibrations, Analytical chemistry

## Abstract

Methods for determination of elements in various types of samples are generally considered to be very precise and highly accurate. For reliable analysis of elements Na, Mg and Ni in food samples is it worthwhile to perform an extensive method validation of high-resolution continuum source flame atomic absorption spectrometry (HR-CS FAAS) according to the principle of pooled calibrations (PoPC). Under routine laboratory conditions of analysis, elevated levels of relative uncertainty of measurement reaching values of more than 50% were detected, which jeopardized the validity of results, even when the measurements in the present study were performed with samples of tap water and borehole water. Comparison of relative uncertainties with corresponding literature results indicates that sample-signal differences may be due to detector noise rather than specimen variations.

## Highlights

• Full correspondence between uncertainties of the results and data of the literature.
• The method validation of the PoPC indicates that Na but not Mg and Ni was determined.
• The LOQ should be replaced with the LLA, owing to estimates of uncertainties.
• Method validations should be performed for all the measurands individually.
• It is proposed that uncertainties are predominantly generated by the apparatus.

## Graphical abstract

**Figure:**
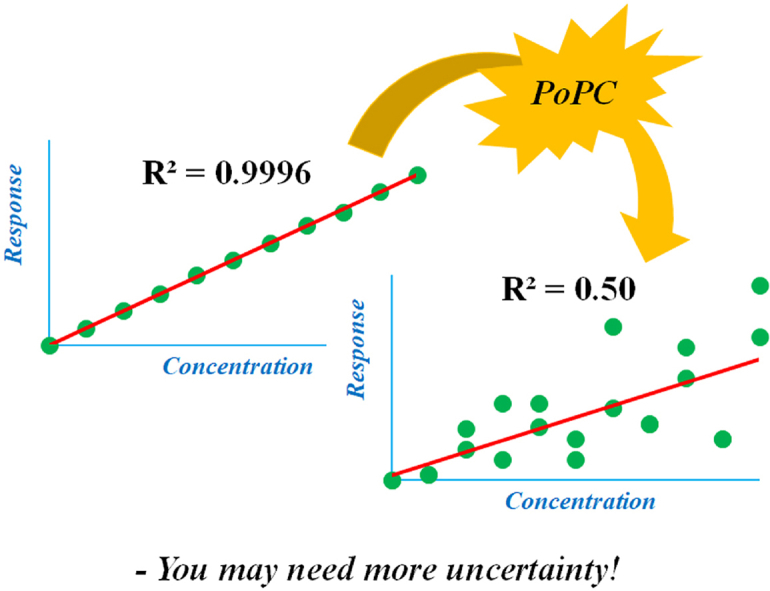

## 1 Introduction

Recently, analytical chemistry realised that striving for low uncertainty, which corresponds to high precision, might not always be synonymous with good quality. New methods of quality assurance (QA) and quality control (QC) are described in guidelines, such as the generic Guide to the Expression of Uncertainty in Measurement (GUM) [1] or the specific Quantifying Uncertainty in Analytical Measurement (QUAM) [2], and they have been adopted by communities within physics and analytical chemistry worldwide. These guidelines focus on the determination of uncertainty of measurement as a tool of making decisions and determine whether the method is fit for purpose [3]. QUAM focuses on quality assurance that is defined as the activities associated with method validations and participation in schemes of proficiency testing [4]. Thus, it indicates that consensus values should be determined for the samples [5], regardless of the method that was used to produce the results. However, the work towards delivery of guidelines that warrants quality of scientific results and compliance with scientific methodology is by no means completed [6]; none of the guidelines of GUM and the International Vocabulary of Metrology (VIM3) [7] mentions the concept of outliers, which has been so extensively promoted by the celebrated guide of ISO 5725 [8]. Merely a brief mention in QUAM [2] reduces the study of influence of outliers on the quality of measurement to a level where it is considered redundant. It was admitted though, that outliers are in fact still important, and they may be involved in the delivery of uncertainties that are largely underestimated, which may be used to explain the concept of ‘dark uncertainty’ [9]. Data that are notoriously present are never seen and never published, which may constitute one of the major paradoxes of contemporary science.

Methods for analysis of trace metals are renowned for their high precision, as it is chemically straightforward to separate those elements from complicated matrices and excellent calibration lines may be obtained from these methods. Hence, a low number of outliers may be expected, and out of 30 references with determination of metals with HR-CS FAAS [[10], [11], [12], [13], [14], [15], [16], [17], [18], [19], [20], [21], [22], [23], [24], [25], [26], [27], [28], [29], [30], [31], [32], [33], [34], [35], [36], [37], [38], [39]], only a single mention of outliers was found [11]. However, in that work, nothing was stated about neither the method of handling them nor of the number of them [11]. Equally interesting, in none of the cited 30 references [[10], [11], [12], [13], [14], [15], [16], [17], [18], [19], [20], [21], [22], [23], [24], [25], [26], [27], [28], [29], [30], [31], [32], [33], [34], [35], [36], [37], [38], [39]] of determination of metals with HR-CS FAAS (Table S1) was used contemporary methods of QA/QC to evaluate the data. In additional publications, only one contribution with analysis of trace elements [40] and two contributions with analysis of sulphur [41,42] were found to even mention the concept of uncertainty that is key to the understanding of QA/QC. Also included in the list of 30 literature references were found methods to analyse Fe and Cu (Table S1), that were used earlier to identify potential errors that could be assigned to issues with lack of training and competencies of staff [43]. In some of the 30 literature references [14,17,36], only a limited amount of information was given with regard to the validation of the method or the method of previous publications was used without further considerations pertaining to the level of uncertainty of measurement [27]. Accordingly, it may thus be suspected that there is scepticism against adopting the methodology of QA/QC [2] within certain areas of analytical chemistry.

In the present investigation, an overwhelming number of outliers was expected to occur after deselecting the software feature where the coefficient of regression is required to be larger than 0.95 (R > 0.95) for the measurements to be accepted for the analysis [43]. With respect to the principle of pooled calibrations (PoPC) [6,44], it is imperative to consider all measured data for the data processing, modeling of uncertainty and statistical analysis. According to the PoPC, all recorded data should be evaluated in the method validation and the uncertainty may be calculated with the *SD* according to the law-of-propagation of uncertainty [2] multiplied by a factor of two [45,46]. Owing to experience from own laboratory, the results of determination of metals in uncomplicated matrices such as borehole water and tap water were associated with unexpected elevated levels of uncertainty [43], as compared with the analyses of food and other types of samples where the contents were determined at lower levels of uncertainties in more complicated matrices [[10], [11], [12], [13], [14], [15], [16], [17], [18], [19], [20], [21], [22], [23], [24], [25], [26], [27], [28], [29], [30], [31], [32], [33], [34], [35], [36], [37], [38]]. By applying the principles of Horwitz [47,48], was considered the worst-possible scenario with respect to uncertainty of measurement, and it was investigated whether the uncertainties could be explained as resulting from either human error [49] or random uncertainty that was generated by the HR-CS FAAS apparatus itself.

## 2 Material and methods

### 2.1 Materials

The standards were prepared by dilution of stock solutions with nitric acid (2% w/w). The nitric acid aqueous solution was prepared by dilution of concentrated nitric acid (70% w/w) with distilled water. The following stock solutions were used for the preparation of standards: Na standard for AAS (Sigma-Aldrich, 05201, Lot BCCB5663, (1000 ± 4) mg^.^L^−1^), Mg standard for AAS (Sigma-Aldrich, 42992, Lot BCCB4545, (1000 ± 5) mg^.^L^−1^) and the Ni stock solution was prepared in-house by dissolving solid nickel acetate tetrahydrate (Ni(CH_3_COO)_2_^.^4H_2_O, Sigma-Aldrich, CAS 6018-89-9, 4.2554 g in 1 L of distilled water) in 2% nitric acid. The standards were freshly prepared for each series of measurements. The standards of Na were prepared (w/v) with the following concentrations 0 mg L^−1^, 0.5 mg L^−1^, 1.0 mg L^−1^, 2.0 mg L^−1^, 3.0 mg L^−1^, 5.0 mg L^−1^, 10 mg L^−1^, and 20 mg L^−1^. Additional standards of Na with concentrations of 40 mg L^−1^, 80 mg L^−1^, and 100 mg L^−1^ with concentrations outside the range with linear responses were used to determine the parameters *A* and *B* of the response curve that was utilised for the calculation of the upper limit of analysis (*ULA*) [50]. For the determination of Mg was used standards with concentrations of 0 mg L^−1^, 0.5 mg L^−1^, 1.0 mg L^−1^, 2.0 mg L^−1^, 3.0 mg L^−1^, 5.0 mg L^−1^, and for Ni, the concentrations of standards were 0 mg L^−1^, 0.5 mg L^−1^, 1.0 mg L^−1^, 2.0 mg L^−1^, 3.0 mg L^−1^, 5.0 mg L^−1^, and 10 mg L^−1^. Also, for Mg and Ni were used standards with concentrations above the linear range of responses and up to 100 mg L^−1^ to determine a value of the *ULA*. No mediators were added to the solutions. The samples of borehole water and tap water were collected locally and used without further pre-treatment.

### 2.2 Instrumentation

The concentrations of sodium (Na), magnesium (Mg) and nickel (Ni) measurands were determined with HR-CS FAAS (AnalytikJena GmbH contrAA® 700) using the wavelengths of 588.9953 nm, 285.2125 nm and 232.0030 nm, respectively. A UPS (Jupiter 15 kVA on-line) was used to stabilise the power supply and to prevent the influence of potential load sheds on the measurements. The incident continuous-source radiation was delivered by a xenon short-arc lamp and the absorbance was measured with an Eschelle-type spectrometer equipped with CCD detector. The solutions were introduced to the flame by an autosampler (AnalytikJena AS-F autosampler), and an air-acetylene flame was used for the atomization of the measurands. The flow rates of air and acetylene were maintained at a ratio of 10:1.

### 2.3 Theory

To further simplify the calculations of uncertainty of measurement, it may be useful to consider the confidence intervals that are provided by the calculations of the MSExcel AnalysisToolPak [51], which yields the following simplified expression for the *RU*:

(1) $$RU≅2∙\left(\frac{s_{b_{0}}}{b_{1}}∙\frac{1}{x}+\frac{s_{b_{1}}}{b_{1}}\right)$$

where *x* is the concentration, $s_{b_{0}}$ is the SD of the intercept, $b_{1}$ is the slope and $s_{b_{1}}$ is the SD of the slope of the calibration line (Table S2) [2]. The factor of two (Eq. (1)) refers to the coverage factor two (*k* = 2) [2]. Thus, the *LLA,* according to the CLs, may be approximated by the following simplified formula:

(2) $$LLA≅\frac{2\;s_{b_{0}}}{b_{1}-2\;s_{b_{1}}}\;if\;b_{1}>2\;s_{b_{1}}$$

In case where $b_{1}<2∙s_{b_{1}}$ (Eq. (2)), it would mean an uncertainty was found to be larger than 100% within the full range of concentrations that were used for the calibrations. Hence, in that case the method should be characterised as not fit for purpose. Also, the *SBR* (Table S2) may be simplified, as follows [43,44]:

(3) $$SBR≅\frac{s_{b_{0}}}{s_{b_{1}}}$$

and finally, the *BRU* may be calculated by the following approximation [43]:

(4) $$BRU≅200∙\frac{s_{b_{1}}}{b_{1}}$$

These formulae are valid for the case of heteroscedasticity with respect to the uncertainties but Eqs. (1)–(4) are valid only when $s_{b_{0}}>0$ within limits of the confidence lines. When $s_{b_{0}}=0$, or if the uncertainties are homoscedastic, then the IUPAC formulae may be used instead to calculate the *LLA* and the *SBR* [43].

## 3 Results and discussion

### 3.1 Data evaluation by the PoPC

With 5–7 data points of the standards was constructed a calibration plot to each series of measurement. A total of 175 data points to Na, 272 data points to Mg and 294 data points to Ni were used to produce the pooled calibrations thus fulfilling the condition of the law-of-large numbers (LLN) [43,52]. For all the three elements, more data were collected by using standards of higher concentrations, in order to determine the values to the constants *A* and *B* of the response function and the concentration of the *ULA* (Table 1) [50]. However, these data to high concentrations were not included in the evaluation of the uncertainty according to the PoPC because they were too few in numbers.

**Table 1:** Figure of merits according to the PoPC. The *LLA* and *SBR* of Mg were not defined because the level of *RU* was larger than 100% and constant for all concentrations. For all the three elements Na, Mg and Ni, the overall levels of uncertainty were very high, as compared with results of literature values (see text). The *RU*s of Horwitz were found to produce values much lower than those of the *BRU*.

Element	Na	Mg	Ni
**Wavelength to measurand (nm)**	588.9953	285.2125	232.0030
**Type of uncertainty**	Heteroscedastic	Heteroscedastic	Heteroscedastic
***LOQ* (mg**^**.**^**L**^**−**^**^1^)**	0.0092	0.050	0.29
***RU*(LOQ) (%)**	14000	1600	380
***A*** [50]	9.2 ± 3.4	10.2 ± 9.5	3.0 ± 1.0
***B* (L**^**.**^**mg**^**−**^**^1^)** [50]	−0.0250 ± 0.0089	−0.026 ± 0.025	−0.0193 ± 0.0067
***LLA* (mg**^**.**^**L**^**−**^**^1^) (Table S1)**	2.25 ± 0.74	–	5.3 ± 1.1
***LLA* (mg**^**.**^**L**^**−**^**^1^)** [1,2]	3.1	2.6	2.5
***LLA* (mg**^**.**^**L**^**−**^**^1^) CL,**Eq. 2	0.59	0.77	0.57
***LLA* (mg**^**.**^**L**^**−**^**^1^)** [47,48]	0.23		
***LLA/LOQ* PoPC**	250	–	10
***SBR* (mg**^**.**^**L**^**−**^**^1^) (Table S1)**	6.8 ± 1.2	–	2.36 ± 0.83
***ULA* (mg**^**.**^**L**^**−**^**^1^) (Table S1)**	27.8 ± 9.9	27 ± 26	36 ± 12
***RU*(*ULA*) (%) (Table S1)**	48	180	86
***RU*(*ULA*) (%)** [47,48]	14	14	13
***BRU (%)* CL,**Eq. 4	9.4	22	12
***BRU* (%) (Table S1)**	44	180	85

**Figure 1:**
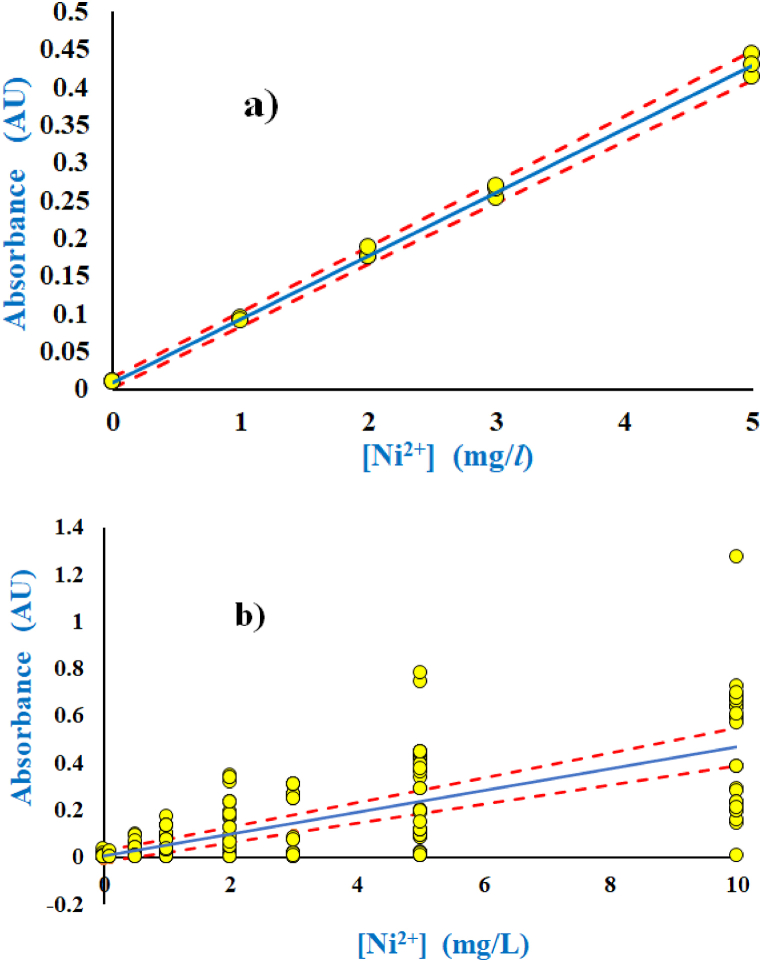
Characteristic example of single regression line of calibration that was prepared on a single day in a single series of experiments. (a) The example refers to the determination of Ni, but similar calibration lines were established for both Na and Mg as well. The equation of the straight line is given by A = (0.0840 ± 0.0023)^.^L^.^mg^−1.^ [Ni^2+^] + (0.0091 ± 0.0063), R^2^ = 0.9977, *n* = 15. The uncertainties are given by the confidence intervals (broken lines), and the slightly positive value to the intercept may be regarded as accidental, owing to the relatively low number of data points. It should be noted that the data of individual concentrations must not be averaged, as that would lead to a loss of information about the uncertainty of measurement. (b) All the straight lines for the operational calibration of Ni were merged to construct a single calibration line with the equation: A = (0.0464 ± 0.0029)^.^L^.^mg^−1^ [Ni ^2+^] + (0.004 ± 0.012), *n* = 294 (full line) and corresponding confidence lines (broken lines), according to the PoPC.

Recent investigations to evaluate the quality of quantification by means of the PoPC [6,45,53,54] suggest that ‘dark data’ or ‘dark uncertainty’ [9] may be involved in the explanation for the lack of concordance with respect to results of analytical chemistry. In terms of food analysis, this worrisome proposition may influence negatively the understanding of the recommended daily intake of minerals [55], nutritional value [56,57] or potential hazards [58]. It is common practice in investigations of analytical chemistry, to construct calibration lines with coefficients of correlations close to one, zero outliers and recovery close to 100%, which were also routinely reported in studies of HR-CS FAAS [[10], [11], [12], [13], [14], [15], [16], [17], [18], [19], [20], [21], [22], [23], [24], [25], [26], [27], [28], [29], [30], [31], [32], [33], [34], [35], [36], [37], [38]]. Indeed, very straight calibration lines were also found for most calibration lines of the present investigation, but the degree of recovery was not considered, as water samples of truly unknown concentrations were used instead to obtain consensus values [5,53,59] thus emphasizing the importance of uncertainty rather than reproducing an already known content. In one series of experiments with three replicates to five standards of nickel (*n* = 15), a straight line was obtained with slope (0.0840 ± 0.0023)^.^L^.^mg^−1^ and *R* = 0.9977 to determine the precise content of nickel in tap water [Ni]_total_ = (10.96 ± 0.18) mg^.^L^−1^ and in borehole water [Ni]_total_ = (0.297 ± 0.081) mg^.^L^−1^ (Fig. 1a). This unexpected and large content of nickel in tap water prompted further investigations. Therefore, a replicate of the experiment (not shown) was performed with *n* = 5 now yielding a calibration line with *R* = 0.9759 and similar slope of (0.087 ± 0.016) L^.^mg^−1^ to determine [Ni]_total_ = (0.00 ± 0.44) mg^.^L^−1^ in tap water and [Ni]_total_ = (−0.39 ± 0.45) mg^.^L^−1^ in borehole water. These concentrations were thus determined below the corresponding LOQ. This major discrepancy between these two results did not lead to removal of the outliers but it spurred a comprehensive analysis of the calibration data with numerous independent series of measurement that were aimed at fulfilling the statistical conditions of the LLN. With 294 data points to the calibration line of the pooled calibrations, and corresponding confidence intervals, the combined results of the analyses are shown for Ni in Fig. 1b, but similar results were obtained for the other two elements Na and Mg as well (not shown). Fig. 1b illustrates the many straight lines of good coefficients of regressions that were merged to yield a single calibration line with much larger spread in the distribution of data (Fig. 1b), as compared to that of individual calibration lines (Fig. 1a). Each calibration line was prepared by least-squares regression of all data combined. Allegedly, this spread of data (Fig. 1b) is supposed to demonstrate the importance of everyday calibrations to correct for the apparatuses' influence on the measured variations of sample concentrations that were determined by the method. As evidenced earlier by numerous examples [6,50,60], this interpretation stands to be disputed, owing to the poor correspondence between the slopes and concentrations of unknowns [61]. This observation was further supported in the present study by the very poor correlations that were found between slopes and the concentrations of elements in water samples. Again, it was confirmed that the uncertainty of measurement, that was derived upon the basis of the PoPC (Fig. 1b), corresponded excellently to the uncertainty of numerous replicates of the samples (Table 2). Although deviations of the *RUs* by approximately a factor of two were found for the results of Na in samples of borehole water and tap water (Table 2), the deviations were negligible in comparison with deviations of *RU*s of samples that were determined by means of single calibration lines, which could amount to an order of magnitude or more, as shown for Ni (above). Numerous investigations of the international measurement evaluation programme (IMEP) convincingly documented the same trend with lack of correspondence between the uncertainty of individual laboratories and that of their reported overall uncertainty of measurement [62,63] but that was explained as originating from lack of training of the laboratory staff. The same conclusion was proposed by Horwitz [47] after evaluating many independent results from the literature. The figure of merits of the PoPC were determined with large uncertainties that indicated large separation between the *LOQ* and the lower limit of analysis (*LLA*), as illustrated by the ratios of *LLA/LOQ* that could be as high as 250 for Na (Table 1). Hence, the PoPC shows that concentrations of Na cannot be reliably determined at concentrations that were close the *LOQ* where the corresponding *RU* would be expected to approach 14000% (Table 1). The *LLA* and start of best range (*SBR*) remained undetermined for Mg because the *RU* never decreased to below 100% at this concentration. The performance of the HR-CS FAAS was not impressive for any of these three elements, as illustrated by the *BRU*'s of 44%, 180% and 85% for Na, Mg and Ni, respectively (Table 1). Thus, there is only vague hope for this particular apparatus, in this particular laboratory setting, to be used to give an indication, as to the content of Na in samples with concentrations close to the *ULA* (Table 1, Table 2). Alas, for the other two elements, no reliable analysis can be expected. Therefore, no further investigations were introduced to determine the influence of potential interferences and the use of mediators. It should be noted that, with this technology of HR-CS FAAS, the *RU*s of the Horwitz formula [47,48] and the *RU* of the CLs were vastly underestimated as the corresponding *RU*s of samples differed by more than an order of magnitude (Table 1, Table 2).

**Table 2:** Comparison of the uncertainties (*U*) and *RU*s of determination of concentrations of the three elements Na, Mg and Ni in water samples of unknown concentrations according to the PoPC. The numbers in brackets provide results that were obtained by including concentrations that were giving values outside the linear range of calibrations.

Sample	Borehole water	Tap water
**[Na**^**+**^**] (mg**^**.**^**L**^**−**^**^1^)**	10 (15)	7.8 (7.3)
***U* (Predicted) (mg**^**.**^**L**^**−**^**^1^)**	7.9	4.4
***U* (Observed) (mg**^**.**^**L**^**−**^**^1^)**	7.1 (43)	4.6 (6.6)
***RU* (Predicted) (%)**	52	61
***RU* (Observed) (%)**	69 (280)	59 (92)
**[Mg**^**2+**^**] (mg**^**.**^**L**^**−**^**^1^)**	4.3	5.8
***U* (Predicted) (mg**^**.**^**L**^**−**^**^1^)**	2.6	11
***U* (Observed) (mg**^**.**^**L**^**−**^**^1^)**	9.1	9.9
***RU* (Predicted) (%)**	180	180
***RU* (Observed) (%)**	210	170
**[Ni]**_**total**_**(mg**^**.**^**L**^**−**^**^1^)**	1.3	7.4
***U* (Predicted) (mg**^**.**^**L**^**−**^**^1^)**	1.9	7.1
***U* (Observed) (mg**^**.**^**L**^**−**^**^1^)**	4.2	16
***RU* (Predicted) (%)**	150	95
***RU* (Observed) (%)**	320	210

### 3.2 Comparison of relative uncertainties (RUs)

**Figure 2:**
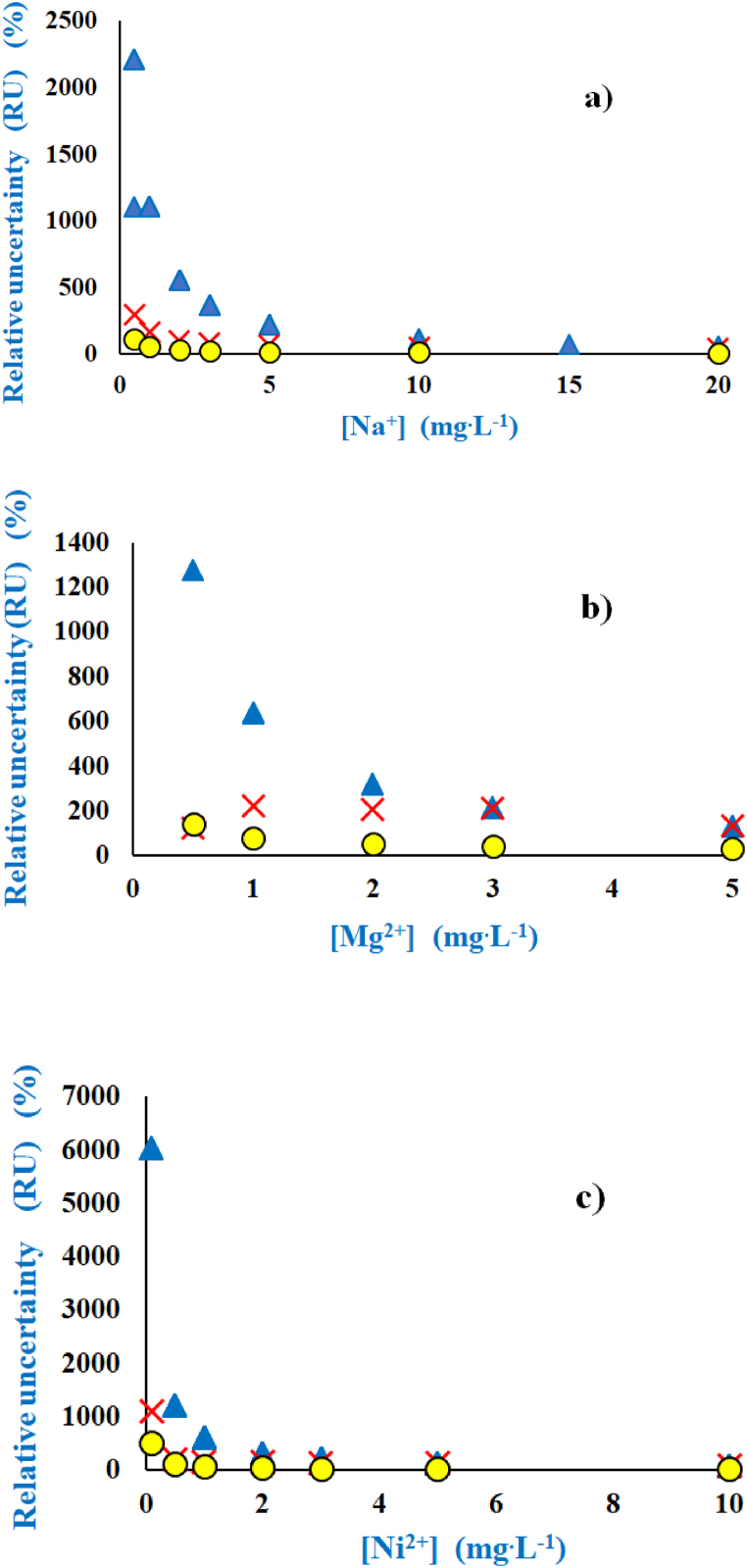
The *RU* depicted as a function of concentrations for (a) Na, (b) Mg and (c) Ni. The *RU* was calculated by three different formulae (▴) IUPAC [43], (●) Confidence lines (CLs) and (✕) PoPC [6,44]. At low concentrations, the *RU*s of the IUPAC formula exceeded the *RU*s of CLs (●) and PoPC (✕) by almost an order of magnitude, and the *RU* of the CLs (●) constituted approximately half the *RU*s of the PoPC (✕).

Three methods of calculations were evaluated for the calculation of the *RU* as a function of the concentration (Fig. 2): The first is the so-called IUPAC method (Fig. 2, ▴) where the uncertainty of measurement depends largely on the magnitude of the standard error that overestimates the *RU* at lower concentrations and underestimate it at larger concentrations, as compared with the second method using *RU*'s of the PoPC (Fig. 2, ✕) and the third method with confidence lines (CLs) (Fig. 2, ●). The correspondence between the *RU*'s of the PoPC and *RU*s of the CLs is excellent and only minor deviations were found at lower concentrations where the *RU*s according to the CLs constituted approximately half the value of the *RU* (PoPC, Fig. 2a–c). The method of using CLs (Fig. 2a–c) is convenient, as they may be calculated by means of the MSExcel Analysis ToolPak that was used to establish the uncertainty of the slopes and intercepts, and they provide figures of merits that are comparable to those of the PoPC (Table 1). Although the method of calculating the RU by means of CLs is convenient and straightforward, it does not provide a deeper understanding of the contributions to the RU that is provided by the PoPC. However, for routine laboratory applications, it may be recommended to characterise the performance of the method by the RUs of the CLs, followed by the method of PoPC whereas the method of IUPAC should be avoided, owing to the issues with overestimating the RU at lower concentrations (Fig. 2).

Recently, the United States Department of Agriculture in the SOP for the pesticide data program (PDP) [64] and the EU regulation 2021/808 [65] recognized the significance of the Horwitz formula [47] to the evaluation of data [64]. These recognitions of the Horwitz formula are probably the first of their kinds, since the results of Horwitz are rarely mentioned in guidelines to QA [[1], [2], [3],^64^]. Horwitz interpreted the unexpected large systematic variations of data as being a result of lack of training of laboratory staff [48]; an idea that was later also adopted by the researchers of the IMEP programme [62,63] and others [49]. The empirical formula of Horwitz considers the variation of all types of data, samples and apparatuses, which is an approach that is expected to produce large uncertainties when the corresponding coefficients of variation (CV) were combined into a universal empirical formula. It may thus be expected that the *RU* of measurement with a single type of apparatus that was used for analysis of one type of sample should be much lower than that predicted by the Horwitz formula [47]. However, recent results indicate that an even larger uncertainty could be established [45,53] when the aim was to obtain compliance between predicted and observed uncertainty, which is fundamental to the fulfilment of scientific methodology.

### 3.3 Collating literature results

To explore the full potential of the PoPC, an analysis of literature data was performed by collecting results of calibrations for each publication and calculating the average value of the concentrations of unknowns, regardless of the type of sample and the setting of the apparatuses. According to the findings above with respect to the poor performance of HR-CS FAAS of the present study, it may be assumed that the differences in concentrations found in the literature originates from uncertainty of measurement only. That is, it was assumed that the observed differences might be not be genuine because none of the methods had been subject to practices and procedures of contemporary method validations [2]. Thus, out of the 30 references that were included in the literature review, from 9 references [10,11,13,28,29,32,34,35,39] was extracted 16 average results for Na, from 12 references [10,11,15,16,22,28,29,32,34,35,37,39] was extracted 14 average results for Mg, and from 5 references [12,13,19,24,34] was extracted 9 average results for Ni (Table S1). The *LOQ*s, slopes, average values and corresponding *RU*s are listed in Table S1 and an overview of the analysis of uncertainty according to PoPC is presented in Table 3. The correspondence is excellent between the *RU*s of slopes, that were found from all the slopes in the literature for each element and the *RU*s of slopes of the present work. The factor of three difference in uncertainty of slopes for the calibration lines for Ni may be regarded as random variation, owing to the relatively low number of data that could be extracted from the literature [12,13,19,24,34]. However, an excellent correspondence was found for the interval of literature slopes and the corresponding interval of slopes that was determined in the present study (Fig. 1b, Table 3). The range of calibration slopes of Na and Mg was not as perfect, but the trend is clear with steeper slopes for Mg, as compared to those of Na (Table 3). The literature values according to *RUs* of samples also displayed an excellent correspondence with those of the present investigation with maximum of a factor of three deviation between the *RU* of Na in tap water and the literature values of the *RU* to Na (Table 3). The analysis of Table 3 demonstrates that the technology of HR-CS FAAS cannot deliver results that are accurate or associated with any reasonable degree of trueness. The method validation of the PoPC has disclosed that the technology HR-CS FAAS delivers calibration lines of good precision, but they cannot correct for the influence of day-to-day variations that are imposed by the apparatus itself. Literature *RU*s (Table S1) that exhibit values lower than the *BRU*s of Table 1 thus indicate that no differences were found between the samples of study and no differences could be discerned according to different settings of the apparatuses. However, even with larger *RU*s it may also be anticipated that no differences were identified because the *RU* depends on the concentration, and samples of the literature might have been analysed at lower concentrations. The findings pertaining to the literature references indicate that there will be issues with the interpretation of the published results, as will be illustrated by the following examples.

**Table 3:** Comparison of *RU*s (%) that were collated from the literature references (Table S2) and the *RU*s (%) using the PoPC of the present study. These results evidence that the variation of literature results can be fully explained by the PoPC. They also show that the variations between concentrations of samples that are found in the literature can be fully explained as random variations within day-to-day measurements.

Element	Na	Mg	Ni
**Literature, *n* (slopes)**	11	10	14
***n (*samples) = *n* (slopes), this work**	30	51	41
**Literature, *n* (samples**)	16	14	9
**Literature average *RU* (%) of slopes**	200	230	59
***RU* (%) of slopes, PoPC, this work**	150	230	190
**Literature slope range (kg**^**.**^**mg**^**−**^**^1^)**	0.0022–1.5025	0.009641–2.4255	0.038–0.08923
**PoPC slope range (kg**^**.**^**mg**^**−**^**^1^), this work**	0.032–0.26	0.027–0.93	0.0011–0.087
***RU* (%) of samples of the literature**	160	110	150
***RU* (%) of samples of borehole water, PoPC, this work**	69 (280)*	210	320
***RU* (%) of samples of tap water, PoPC, this work**	59 (92)*	170	210

*Two concentrations of borehole water and two results of tap water were determined outside the linear range of calibrations. The numbers in brackets show the values that were obtained if those ‘outliers’ were included in the calculations.

In one example with analysis of juices where the *RU*s of Na and Mg were calculated to be close to the average *RU*s of the literature references combined (Table S1), superior accuracy of −4.6%–5.7% was reported but no evidence was presented for those low percentages [11]. Although it may be not reasonable to compare the contents of minerals in ground coffee and brewed coffee, much less variation was found in samples of brewed coffee (*RU* = 44%) than those of the ground coffee (*RU* = 480%) [35]. That may be not surprising because of potentially large variation between the brands of coffee but the results indicate that there is no correlation between the contents of Na and Mg in ground coffee and brewed coffee, which is more surprising. In addition, more sodium was found in 6 out of 10 samples of brewed coffee as compared with the content of ground coffee, which should have also raised cause of concern [35]. Further, the *RU*s of 180% and 42% for Na and Mg, respectively, in samples of expresso may not suffice as evidence for differences that were related to the country of origin, according to the PoPC (Table 3). Similar homogeneous results of Mg (*RU* = 34%) were found in brews of instant coffees and, among the elements analysed, Mg was the element of highest precision and with the highest bio-accessibility [15]. That conclusion is contrary to the present findings where Mg was found to be the element with the lowest precision (Table 1, *BRU* = 180%). With respect to samples of brewed tea, issues with low concentrations led to introduction of chemometric methods to calculate concentrations. However, it was not made clear if the different types of tea were measured on the same day or on different days where the apparatus had been switched off and switched on between each series of measurement [13]. According to the present findings of the PoPC, some very large *RU*s were encountered for samples with concentrations close to the *LOQ*, which could easily explain the large overall *RU* for both Na and Ni in the samples of tea (Table S1) [13]. The same trend was found for determination of Na and Mg in infant formulas [28] where the overall *RU*s of 177% for Na and 83% for Mg were almost identical with the average values of *RU*s of 160% and 110%, respectively, of the collated results of the literature (Tables S1 and 3). In dairy foods, a factor of ten higher concentration of Ca in comparison to Mg is likely to be correctly assessed by the method, but even such a result may be challenged when it is not exactly clear at which concentration the samples were measured [22]. The linear range is often very narrow and the maximum concentration may be very low [10,20,26,30,31,34,38], as in the analysis of commercial squids [38], where Na was analysed within the range of 0.1 mg L^−1^ to 1.20 mg L^−1^ and Mg within 0.05 mg L^−1^ to 0.35 mg L^−1^, which are concentrations well below those of the corresponding *LLAs* (Table 1). According to these examples (above), there is a plethora of issues with results that have not been subjected to contemporary methods of QA/QC. Although the precision was excellent, concerns should be raised with respect to data interpretations and trueness of the results.

From the data of Table S1, it may be observed that *RU*s of the literature *LOQ*s were determined as 450%, 260% and 470% for Na, Mg and Ni, respectively, which shows that the combination of SD of background noise level and differences in slopes leads to *LOQ*s that are not reliable. According to the corresponding average values of the *LOQ*s (Table S1), the *RU*s of the PoPCs were determined as 250%, 590% and 490%, respectively. These latter values did not differ by more than a factor of two from the *RU*s of the literature *LOQ*s, which is quite satisfactory when considering the large levels of uncertainty of such low concentrations. The *RU*s of samples (Table 3) did not appear random to the same degree, which evidences that the HR-CS FAAS delivers results that are deceptive in the sense that there is a large spread of results between days that cannot be attributed to neither lack of competence of the operators nor matrix effects, but it should be attributed to properties of the apparatuses. It may be proposed that the uncertainty of measurement originates predominantly from long-term drift phenomena of the detector [50]. In none of the cited references appears the concept of ‘uncertainty’, and none of the references applied contemporary methods of QA/QC [1,2] to their analyses. Hence, the more surprising is it that recoveries of 100% were reported after spiking with the elements for analysis of CRMs in all of them, which remains one of the main paradoxes of the combined investigations. It is difficult to understand that no efforts regarding QA/QC can lead to such excellent results. Thus, more detailed investigations are needed with respect to detectors and software of the apparatuses. Such efforts may also include close collaboration with the manufacturers of the apparatuses, who can assist with testing of, e.g., the performance of the detectors. Compelling evidence was provided to suggest that HR-CS FAAS can deliver qualitative results whereas it cannot be used for quantification of Na, Mg and Ni in these types of samples. Therefore, it may be suggested that the contents of Na, Mg and Ni, that were determined in samples [[11], [12], [13],^15^,^16^,^18^,[21], [22], [23], [24],[27], [28], [29], [30],^34^,^35^,[37], [38], [39]] of food and other types of samples [10,14,17,19,20,25,26,31,32,36] should be compared with results produced by auxiliary technologies because the results, that were presented in those literature references may not be reliable or they may be regarded as incomplete.

## 4 Conclusion

Compelling evidence was provided to demonstrate that HR-CS FAAS can deliver qualitative results whereas it cannot be used to quantification of Na, Mg and Ni in any type of sample. At concentrations close to the upper limit of analysis (*ULA*), it would be possible to analyse for contents of Na in the samples, but the concentrations of Mg and Ni remained undetermined. The results indicate that a full method validation should be performed for every single element and incorporating all concentrations of the calibration line to obtain the relevant figures of merits that characterises the true performance of the method. By applying the PoPC to the evaluation of the data, it was confirmed, in relation to earlier investigations, that the limit of quantification (*LOQ*) should be replaced with the concept of the lower limit of analysis (*LLA*) to estimate the degree of trueness that is associated with the lower concentrations of the method. Therefore, it may be postulated that the contents of Na, Mg and Ni, that were determined in samples of food and other types of samples should be assigned with uncertainties that correspond to the predictions of the PoPC. The present investigation demonstrates the first example of full correspondence between predicted uncertainties, sample uncertainties and uncertainties of the literature. Accordingly, complete statistical control was obtained. It is proposed that this type of analysis of the uncertainty of measurement should be performed for all methods of analytical chemistry, but it may also be applied to other areas of science where it is important to obtain quantitative information.

The elevated levels of uncertainties may be explained by drift phenomena of the detector that require further investigations to identify and quantify them in more detail.

## Author contribution statement

Jens Enevold Thaulov Andersen: Conceived and designed the experiments; Analysed and interpreted the data; Wrote the paper.

Keaboletse Moemedi, Kebabonye Katse: Conceived and designed the experiments; Performed the experiments; Analysed and interpreted the data; Contributed reagents, materials, analysis tools or data.

## Funding statement

This research did not receive any specific grant from funding agencies in the public, commercial, or not-for-profit sectors.

## Data availability statement

Data will be made available on request.

## Declaration of interest's statement

The authors declare no conflict of interest.

## Abbreviations

Quality assurance (QA), Quality control (QC), High-resolution-continuous-source flame atomic absorption spectrometry (HR-CS FAAS), limit of quantification (LOQ), standard deviation (SD), confidence lines (CLs), Coefficient of variation (CV), principle of pooled calibrations (POPC), law-of-large numbers (LLN), Lower-limit of analysis (LLA), start of best range (SBR), upper limit of analysis (ULA), relative uncertainty (RU), best-relative uncertainty (BRU).

## Appendix B Supplementary data

Table S1 R2 Table S1 R2

Table S2 R2 Table S2 R2
