# Supplementary material for: The principle of pooled calibrations delivers full correspondence between uncertainties of measurements of Na, Mg and Ni when determined using HR-CS FAAS
Source: Heliyon. 2023 Feb 8;9(2):e13562. doi: 10.1016/j.heliyon.2023.e13562 (PMC9947278; doi:10.1016/j.heliyon.2023.e13562)
Supplement: Table S1 R2 [file mmc1.docx]

**Table S1**. Literature survey of the method of HR-CS FAAS that was used to determine the contents of Na, Mg and Ni in

samples of food, beverages, diesel, gasoline, steel and soil. The empty cells indicate that information is missing in the literature references. The LOQs and the slopes were listed as figures of merits but the coefficients of regression for the calibration lines were not included, as they were very close to one in all the publications. For the majority of references, no outliers were reported, and the degree of recovery of analyte added to the samples was always close to 100 %. The sample RU refers to the RU that was calculated by using all the samples in each publication with no regard to neither the treatment of the sample nor the settings of the apparatus.

| First author | Reference | Matrix | Element | Na LOQ (mg/kg) | Mg LOQ (mg/kg) |
| --- | --- | --- | --- | --- | --- |
| dos Santos | 10 | B7-diesel oil | Ni |  |  |
| Pohl | 11 | Apple Juices | Na, Mg | 10 | 2.5 |
| Leite | 12 | Ethanol fuel | Na, Ni | 300 |  |
| Brandao | 13 | Fortified milk powder | Fe |  |  |
| Brandao | 14 | Dairy products | Mg |  | 640 |
| Pohl | 15 | Functional apple beverages | Mg |  | 12 |
| Nunes | 16 | Vegetable oil | Ni |  |  |
| Baytak | 17 | Almond bark | Ni |  |  |
| Filho | 18 | Lubricating oil treatments | Ni |  |  |
| Filho | 18 | Lubricating oil treatments | Ni |  |  |
| Filho | 18 | Lubricating oil treatments | Ni |  |  |
| Filho | 18 | Lubricating oil treatments | Ni |  |  |
| Yilmaz | 19 | Tissues of tench | Fe, Cu |  |  |
| Gamela | 20 | Infant formulas | Na, Mg | 5700 | 20 |
| Gamela | 20 | Infant formulas | Na, Mg |  |  |
| Zambrzycka-Szelewa | 21 | Polish Beers | Na, Mg | 40 | 80 |
| Trindade | 22 | Vegetable oil | Ni |  |  |
| Santos | 23 | Baby leaf vegetable | Na, Mg | 0.35 | 0.21 |
| de Jesus | 24 | Trace elements in biodiesel | Na, Mg | 260 | 110 |
| Guida | 25 | Micronutrients | Na, Mg | 130 | 90 |
| Raposo Jr | 26 | Distilled alcoholic beverages | Cu |  |  |
| Oliveira | 27 | Instant coffee and coffee substitutes | Na, Mg | 850 | 220 |
| Özdestan | 28 | Turkish coffee | Na, Mg | 850 | 220 |
| Peña-Vázquez | 29 | Metals in seawater and waste water | Ni |  |  |
| Peña-Vázquez | 29 | Metals in seawater and waste water | Ni |  |  |
| Peña-Vázquez | 29 | Metals in seawater and waste water | Ni |  |  |
| Miranda | 30 | Foodstuff | Na, Mg | 30 | 50 |
| Miranda | 30 | Foodstuff | Na, Mg | 60 | 30 |
| Torrinha | 31 | Commercial squids | Na, Mg | 300 | 300 |
| Paz-Rodríguez | 32 | Tea | Na, Ni |  |  |
| Paz-Rodríguez | 32 | Tea | Na, Ni | 119 |  |
| Paz-Rodríguez | 32 | Tea | Na, Ni | 137 |  |
| Paz-Rodríguez | 32 | Tea | Na, Ni | 195 |  |
| Paz-Rodríguez | 32 | Tea | Na, Ni | 850 |  |
| Júnior | 33 | Soil extracts | Fe, Cu |  |  |
| Stelmach | 34 | Instant coffee brews | Mg |  | 5.7 |
| Oliveira | 35 | Plant leaves | Mg |  | 600 |
| Leite | 36 | Brazilian automotive gasoline | Fe, Cu |  |  |
| Fernández-López | 37 | Alcohol and non-alcoholic drinks | Fe, Cu |  |  |
| Nakayama | 38 | Steel | Ni |  |  |
| Oliveira | 39 | Espresso beverages of pure origin coffee | Na, Mg |  |  |
| Average |  |  |  | 614 | 159 |
| Standard deviation | |  |  | 1389 | 209 |
| RU |  |  |  | 452 | 263 |

Table S1 (continued)

| First author | Reference | Ni LOQ (mg/kg) | Na slope (AU/(mg/kg)) | Mg slope (AU/(mg/kg)) | Ni slope (AU/(mg/kg)) | Na range (mg/kg) | Mg range (mg/kg) |
| --- | --- | --- | --- | --- | --- | --- | --- |
| dos Santos | 10 | 40 |  |  | 0.038 |  |  |
| Pohl | 11 |  |  |  |  |  |  |
| Leite | 12 | 140 | 0.4277 |  | 0.04096 | 0.25-1.0 |  |
| Brandao | 13 |  |  |  |  |  |  |
| Brandao | 14 |  |  | 0.01363 |  |  | 1.0-10 |
| Pohl | 15 |  |  |  |  |  |  |
| Nunes | 16 | 1900 |  |  | 0.058 |  |  |
| Baytak | 17 |  |  |  | 0.059 |  |  |
| Filho | 18 | 17 |  |  | 0.08526 |  |  |
| Filho | 18 | 47 |  |  | 0.05499 |  |  |
| Filho | 18 | 88 |  |  | 0.04663 |  |  |
| Filho | 18 | 100 |  |  | 0.04227 |  |  |
| Yilmaz | 19 |  |  |  |  |  |  |
| Gamela | 20 |  | 0.0022 | 0.3091 |  | 25-320 | 0.15-1.6 |
| Gamela | 20 |  |  |  |  |  |  |
| Zambrzycka-Szelewa | 21 |  | 0.0127 | 0.0729 |  | 1-66 | 1.0-12 |
| Trindade | 22 | 210 |  |  | 0.0436 |  |  |
| Santos | 23 |  | 0.677 | 1.042 |  | 0.05-0.75 | 0.02-0.45 |
| de Jesus | 24 |  | 1.5025 | 2.4255 |  | 0.02‑0.14 | 0.06‑0.42 |
| Guida | 25 |  | 0.717 | 0.689 |  | 0.2-5.0 | 0.2-4.0 |
| Raposo Jr | 26 |  |  |  |  |  |  |
| Oliveira | 27 |  | 0.151 | 1.014 |  | 0.350–6.200 | 0.080–1.100 |
| Özdestan | 28 |  | 0.002327 | 0.009641 |  | 0.5–2.5 | 0.2–1.0 |
| Peña-Vázquez | 29 | 67 |  |  | 0.0594 |  |  |
| Peña-Vázquez | 29 |  |  |  | 0.069 |  |  |
| Peña-Vázquez | 29 |  |  |  | 0.0707 |  |  |
| Miranda | 30 |  |  |  |  |  |  |
| Miranda | 30 |  |  |  |  |  |  |
| Torrinha | 31 |  | 0.6417 | 1.2728 |  | 0.10 – 1.20 | 0.05 – 0.35 |
| Paz-Rodríguez | 32 |  |  |  |  |  |  |
| Paz-Rodríguez | 32 | 55 | 0.363 |  | 0.087 |  |  |
| Paz-Rodríguez | 32 | 50 | 0.342 |  | 0.089 |  |  |
| Paz-Rodríguez | 32 | 55 |  |  |  |  |  |
| Paz-Rodríguez | 32 | 56 |  |  |  |  |  |
| Júnior | 33 |  |  |  |  |  |  |
| Stelmach | 34 |  |  |  |  |  |  |
| Oliveira | 35 |  |  |  |  |  | 5.0-40 |
| Leite | 36 |  |  |  |  |  |  |
| Fernández-López | 37 |  |  |  |  |  |  |
| Nakayama | 38 |  |  |  |  |  |  |
| Oliveira | 39 |  |  |  |  |  |  |
| Average |  | 217 | 0.44 | 0.761 | 0.0603 |  |  |
| Standard deviation |  | 508 | 0.4436 | 0.7891 | 0.0177 |  |  |
| RU |  | 467 | 202 | 207 | 59 |  |  |

Table S1 (continued)

| First author | Reference | Ni range (mg/kg) | n(calibration) | Any outliers? | Sample average Na (mg/kg) | Sample RU% Na | Sample average Mg (mg/kg) |
| --- | --- | --- | --- | --- | --- | --- | --- |
| dos Santos | 10 | 1.0-4.0 | ? | No |  |  |  |
| Pohl | 11 |  | 5 | Yes | 17 | 105 | 41 |
| Leite | 12 | 0.5-2.0 | 5 | No |  |  |  |
| Brandao | 13 |  | ? | No |  |  |  |
| Brandao | 14 |  | ? | No |  |  | 0.369 |
| Pohl | 15 |  | ? | No |  |  |  |
| Nunes | 16 |  | ? | No |  |  |  |
| Baytak | 17 |  | 5 | No |  |  |  |
| Filho | 18 | 0.01-1.0 | ? | No |  |  |  |
| Filho | 18 |  | ? | No |  |  |  |
| Filho | 18 |  | ? | No |  |  |  |
| Filho | 18 |  | ? | No |  |  |  |
| Yilmaz | 19 |  | ? | No |  |  |  |
| Gamela | 20 |  | ? | No | 2995 | 177 | 390 |
| Gamela | 20 |  | ? | No | 67 | 170 | 20 |
| Zambrzycka-Szelewa | 21 |  | ? | No | 36 | 106 | 115 |
| Trindade | 22 |  | ? | No |  |  |  |
| Santos | 23 |  | 5 | No | 59 | 140 | 61 |
| de Jesus | 24 |  | ? | No | 1.2 | 100 |  |
| Guida | 25 |  | ? | No | 86 | 186 | 290 |
| Raposo Jr | 26 |  | 10 or 2 | No |  |  |  |
| Oliveira | 27 |  | >-5 | No | 157 | 140 | 206 |
| Özdestan | 28 |  | 6 with three replicates | No | 568 | 502 | 1123 |
| Peña-Vázquez | 29 |  | 5 | No |  |  |  |
| Peña-Vázquez | 29 |  | ? | No |  |  |  |
| Peña-Vázquez | 29 |  | ? | No |  |  |  |
| Miranda | 30 |  | ? | No | 1646 | 218 | 1303 |
| Miranda | 30 |  | ? | No |  |  |  |
| Torrinha | 31 |  | n>-5 | No |  |  |  |
| Paz-Rodríguez | 32 |  | ? | No | 2.1 | 310 |  |
| Paz-Rodríguez | 32 |  | ? | No | 0.24 | 98 |  |
| Paz-Rodríguez | 32 |  | ? | No | 0.074 | 84 |  |
| Paz-Rodríguez | 32 |  | ? | No | 2.2 | 23 |  |
| Paz-Rodríguez | 32 |  | ? | No | 9.5 | 24 |  |
| Júnior | 33 |  | ? | No |  |  |  |
| Stelmach | 34 |  | 5 | No |  |  | 95 |
| Oliveira | 35 |  | ? | No |  |  | 2.7 |
| Leite | 36 |  | ? | No |  |  |  |
| Fernández-López | 37 |  | 1 | No |  |  |  |
| Nakayama | 38 |  | ? | No |  |  |  |
| Oliveira | 39 |  | ? | No | 28 | 180 | 1200 |
| Average |  |  |  |  | 355 | 160 | 373 |
| Standard deviation |  |  |  |  | 818 | 116 | 492 |
| RU |  |  |  |  | 461 | 145 | 264 |

Table S1 (continued)

| First author | Reference | Sample RU% Mg | Sample average Ni (mg/kg) | Sample RU% Ni | Recovery ~100 % ? |
| --- | --- | --- | --- | --- | --- |
| dos Santos | 10 |  |  |  | Yes |
| Pohl | 11 | 83 |  |  | Yes |
| Leite | 12 |  |  |  | Yes |
| Brandao | 13 |  |  |  | Yes |
| Brandao | 14 | 189 |  |  | ? |
| Pohl | 15 |  |  |  | Yes |
| Nunes | 16 |  | 1.7 | 134 | Yes |
| Baytak | 17 |  |  |  | Yes |
| Filho | 18 |  |  |  | Yes |
| Filho | 18 |  |  |  | Yes |
| Filho | 18 |  |  |  | Yes |
| Filho | 18 |  |  |  | Yes |
| Yilmaz | 19 |  |  |  | ? |
| Gamela | 20 | 83 |  |  | No |
| Gamela | 20 | 164 |  |  | No |
| Zambrzycka-Szelewa | 21 | 41 |  |  | ? |
| Trindade | 22 |  | 0.26 | 37 | Yes |
| Santos | 23 | 77 |  |  | Yes |
| de Jesus | 24 |  |  |  | Yes |
| Guida | 25 | 97 |  |  | Yes |
| Raposo Jr | 26 |  |  |  | Yes |
| Oliveira | 27 | 146 | 0.075 | 297 | Yes |
| Özdestan | 28 | 186 |  |  | Yes |
| Peña-Vázquez | 29 |  |  |  | Yes |
| Peña-Vázquez | 29 |  |  |  | Yes |
| Peña-Vázquez | 29 |  |  |  | Yes |
| Miranda | 30 | 180 |  |  | Yes |
| Miranda | 30 |  |  |  | Yes |
| Torrinha | 31 |  |  |  | ? |
| Paz-Rodríguez | 32 |  | 67 | 345 | Yes |
| Paz-Rodríguez | 32 |  | 39 | 58 | Yes |
| Paz-Rodríguez | 32 |  | 162 | 233 | Yes |
| Paz-Rodríguez | 32 |  | 19 | 92 | Yes |
| Paz-Rodríguez | 32 |  | 29 | 120 | Yes |
| Júnior | 33 |  |  |  | Yes |
| Stelmach | 34 | 34 |  |  | Yes |
| Oliveira | 35 | 50 |  |  | Yes |
| Leite | 36 |  |  |  | Yes |
| Fernández-López | 37 |  |  |  | ? |
| Nakayama | 38 |  | 0.16 | 61 | Yes |
| Oliveira | 39 | 42 |  |  | Yes |
| Average |  | 106 | 35 | 153 |  |
| Standard deviation |  | 59 | 53 | 112 |  |
| RU |  | 112 | 299 | 147 |  |
