# Supplementary material for: The principle of pooled calibrations delivers full correspondence between uncertainties of measurements of Na, Mg and Ni when determined using HR-CS FAAS
Source: Heliyon. 2023 Feb 8;9(2):e13562. doi: 10.1016/j.heliyon.2023.e13562 (PMC9947278; doi:10.1016/j.heliyon.2023.e13562)
Supplement: Table S2 R2 [file mmc2.docx]

**Table S2.** List of equations to the PoPC that were used to evaluate the data for determination of Na, Mg and Ni in samples of borehole water and tap water by means of HR-CS FAAS. The notation of QUAM appendix E [2] was used to derive the equations that are re-written and simplified versions of earlier publications. The listed equations refer to the heteroscedastic case [43].

| Abbreviation | Explanation | Equation |
| --- | --- | --- |
| *y* | Response values to the linear-least squares regression line of calibration | $y=b_{0}+b_{1}\cdot x$ |
| *u(x)* | Standard deviation of the calibration line | $u\left( x \right)\cong s_{0}^{'}+s_{1}^{'}\cdot x$ |
| $B_{0}$ | Parameter of the law-of-propagation of uncertainty related to standard deviations of intercepts | $B_{0}=\frac{{s_{0}^{'}}^{2}+s_{b_{0}}^{2}}{2\cdot b_{1}^{2}}$ |
| $B_{1}$ | Parameter of the law-of-propagation of uncertainty related to standard deviations of *u(x)* | $B_{1}=\frac{s_{0}^{'}\cdot s_{1}^{'}}{b_{1}^{2}}$ |
| $B_{2}$ | Parameter of the law-of-propagation of uncertainty related to standard deviations of slopes | $B_{2}=\frac{{s_{1}^{'}}^{2}+s_{b_{1}}^{2}}{2\cdot b_{1}^{2}}$ |
| *RU* | The relative uncertainty of the calibration line and samples. It is equal to the standard deviation multiplied by a factor of two when the law-of-large numbers is fulfilled. | $RU=200\cdot\sqrt{B_{2}+\frac{B_{1}}{\bar{x}}+\frac{B_{0}}{\bar{x}^{2}}} \%$ |
| *LLA* | Lower limit of analysis. The concentration where the relative uncertainty is 100% | $LLA=\frac{B_{1}+\sqrt{B_{1}^{2}+4\cdot\left( {\frac{1}{4}-B}_{2} \right)\cdot B_{0}}}{2\cdot\left( {\frac{1}{4}-B}_{2} \right)}$ |
| *SBR* | Start of best range. The concentration at which the *RU* becomes constant | $SBR=\frac{B_{1}+\sqrt{B_{1}^{2}+4\cdot{B_{0}\cdot B}_{2}}}{2\cdot B_{2}}$ |
| *ULA* | Upper limit of analysis of the response function with intercept $x_{0}$ and decay factor *B* (*B* < 0) | $ULA=x_{0}-\frac{1}{B}\cdot ln2$ |
| *RU* | Relative uncertainty that applies to both standards and unknowns | $RU\left( FOM \right)=200\cdot\sqrt{B_{2}+\frac{B_{1}}{\bar{x}}+\frac{B_{0}}{\bar{x}^{2}}} \%$ |
| *BRU* | Figure of merit. Best relative uncertainty that can possibly be achieved by the method | $BRU \underset{\to}{\bar{x}\to\infty} 200\cdot\sqrt{B_{2}} \%$ |
